# Oral Microbiome and Metabolome Changes During Orthodontic Treatments: A Systematic Review of Limited Clinical Evidence

**DOI:** 10.3390/medicina62010224

**Published:** 2026-01-21

**Authors:** Michela Boccuzzi, Riccardo Aiuto, Leonardo Lombardo, Matteo Piasente, Andrea Edoardo Bianchi, Alberto Clivio

**Affiliations:** Italian Stomatologic Institute, Via Pace 21, 20122 Milan, Italy; riccardo.aiuto@alice.it (R.A.); leonardo.lombardo@hotmail.it (L.L.); matteo.piasente@gmail.com (M.P.); andrea.ebianchi@gmail.com (A.E.B.); aclivio@isimilano.eu (A.C.)

**Keywords:** microbiome, metabolome, orthodontic appliances, clear aligners, fixed appliances

## Abstract

*Background and Objectives*: Recent advances in dentistry include microbiological and metabolomic analyses, which have the potential to improve the understanding of oral microbiome–host imbalances during orthodontic treatment. Fixed appliances, functional devices and, more recently, clear aligners have been associated with several oral health conditions, including enamel demineralization, dental caries, gingivitis, periodontitis and root and bone resorption. In this context, metabolomic approaches may enable the identification of metabolites in biological samples that could potentially serve as biomarkers and reflect functional biological changes within the oral ecosystem. Investigating orthodontic appliances and associated metabolomic alterations may therefore contribute to advancing current knowledge in orthodontics. This systematic review aimed to describe the available evidence on oral metabolomic changes during orthodontic treatment. *Materials and Methods*: A systematic literature search was conducted in PubMed, Web of Science, Scopus and the Cochrane Library. A total of 1632 records were identified. After duplicate removal and screening, 18 full-text articles were assessed for eligibility. Of these, 15 studies were excluded, and three studies met the inclusion criteria. Risk of bias was assessed using the ROBINS-I and RoB 2 tools, and the GRADE approach was applied to evaluate the certainty of evidence. The review protocol was registered in PROSPERO (CRD420251141544). *Results*: Three studies met the inclusion criteria. Overall, the available evidence was limited and heterogeneous. The included studies suggested potential differences in oral microbiome composition and metabolomic profiles between patients treated with fixed appliances and those treated with clear aligners. Reported metabolomic findings were exploratory and involved amino acid-related, immune-associated, and acidic metabolic pathways. Limitations: Only three studies were included, all conducted in a single country. The small sample size and methodological heterogeneity limit the generalizability of the findings. In addition, potential confounding variables highlight the need for further standardized longitudinal studies.

## 1. Introduction

Within the framework of omics sciences, metabolomics represents a relatively recent research approach in dentistry and, more recently, in orthodontics. It focuses on the qualitative and quantitative analysis of low-molecular-weight compounds present in biological samples and associated with several physiological and pathological conditions [1]. Unlike genomic or microbiome analyses, metabolomics captures downstream metabolic activity and reflects the dynamic interactions among host tissues, microorganisms, and environmental factors.

Current evidence reports heterogeneous findings regarding changes in the oral microbiota associated with orthodontic treatments.

Several studies have identified *Streptococcus* species in patients treated with clear aligners, together with *Fusobacterium*, potentially influenced by aligner material properties [2,3].

In contrast, fixed appliance therapy has been associated with the presence of *Tannerella*, *Actinomyces*, *Prevotella*, *Porphyromonas gingivalis* and *Streptococcus* species [4,5].

However, Lombardo et al. reported overlapping microbial profiles between clear aligners and fixed appliances, with a higher relative abundance of *Fusobacterium nucleatum* in patients undergoing fixed orthodontic treatment [6].

The oral ecosystem is influenced by multiple endogenous and exogenous factors, including systemic health status, oral hygiene practices, pharmacological therapies, and orthodontic appliances, all of which may contribute to metabolic alterations.

Oral metabolites, representing substrates, intermediates, or end products of specific metabolic pathways, reflect the host’s dynamic biological response and have been associated with clinical conditions such as gingivitis, periodontitis, dental caries, and white spot lesions [7].

Previous studies have shown that acidic metabolites derived from microbial sugar fermentation contribute to local pH reduction and may promote enamel demineralization [8].

Among these, 5 aminopentanoic acid has been reported to be associated with tissue necrosis [9,10].

Alterations in amino acid profiles have been described in patients with poor oral hygiene, while changes suggestive of increased immune-related metabolic activity have been observed in orthodontic patients undergoing aligner treatment, resembling patterns reported in periodontal disease [11,12].

Lipid-related metabolites have also been associated with dental caries [13].

Additionally, elevated levels of putrescine, ornithine, hypoxantine, 4-hydroxycinnamic acid, and phosphoethanolamine have been described to be associated with periodontal inflammation [14].

By reflecting phenotypic expression at the molecular level, metabolomics enables the identifications of potential biomarkers that may support improved prognostic assessment, monitoring of inflammatory processes and tissue remodeling, and evaluation of orthodontic treatments in terms of oral health outcomes across different patient subgroups [15].

Metabolomics analyses typically involve analytical techniques such as chromatography, including liquid chromatography (LC) and gas chromatography (GC), coupled with mass spectrometry (MS), as well as nuclear magnetic resonance (NMR).

These approaches can be classified as targeted or untargeted, depending on whether the objective is the quantification of predefined metabolites or the comprehensive profiling of metabolic patterns within a biological sample [16,17,18,19,20].

In this context, the assessment of oral metabolites may provide an instantaneous and functional snapshot of the biological status of the oral ecosystem, complementing existing microbiome-based evidence in orthodontic patients.

The aim of this systematic review is to explore and summarize the available evidence on oral metabolomic changes reported during orthodontic treatment, with the purpose of identifying metabolites of potential interest.

## 2. Materials and Methods

### 2.1. Protocol Registration

This systematic review was conducted in accordance with the methodological guidelines of the Cochrane Handbook for Systematic reviews and is reported following the PRISMA statement. The review protocol was registered in the PROSPERO International Prospective Register of Systematic Reviews on 5 September 2025 (registration number: CRD420251141544) [21]. The Supplementary Material can be found at PRISMA 2020 Checklist.

### 2.2. Eligibility Criteria

The review question was formulated according to the PICO framework as follows:

- P(population): patients of any age.
- I(interventions): orthodontic treatments.
- C(comparison): different types of orthodontic appliances (brackets, clear aligners and functional appliances).
- O(outcome): identification of metabolites.

The inclusion criteria were as follows:

(1) Studies published in English.
(2) No restriction on publication date.
(3) Clinical studies investigating the association between orthodontic treatment and metabolomic analysis, without restrictions on study design. This included cross-sectional, retrospective, and prospective studies. No predefined age restrictions were applied during study selection.

The exclusion criteria considered were

(1) In vitro studies.
(2) Animal model studies.
(3) Studies involving periodontally compromised patients.
(4) Studies including patients with systemic conditions.
(5) Studies involving patients undergoing pharmacological treatments.

### 2.3. Information Sources and Search Strategy

A comprehensive literature search was conducted in the following electronic databases: PubMed, Web of Science, Scopus and the Cochrane Library. The search strategy combined keywords using Boolean operators to identify eligible studies: (metabolomic OR metabolome OR “metabolic profiling”) AND (microbiome OR microbiota) AND (orthodontics OR “fixed appliance” OR “clear aligner” “OR aligner” OR “functional appliance”). The reference lists of the included studies were manually screened to identify additional relevant articles. An initial exploratory search was conducted in August 2025 to assess the availability of evidence on this emerging topic in orthodontics. The formal systematic literature search was independently conducted by two reviewers (M.B. and M.P.) on 1 September 2025, across all selected databases. Study screening and selection procedures were subsequently carried out between 1 September and 29 September 2025.

### 2.4. Study Selection

**Figure 1:**
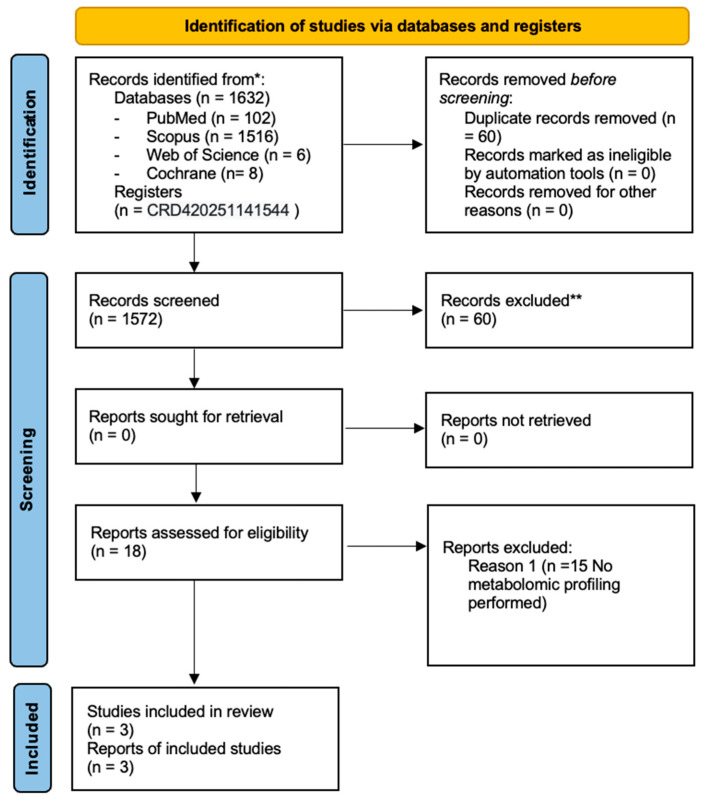
Prisma flow diagram.

A total of 1632 articles were identified. Sixty duplicates were removed by two independent reviewers (M.B and M.P.) using Rayyan (Qatar Computing Research Institute, Doha, Qatar; https://www.rayyan.ai, accessed on 3 September 2025). Subsequently, 1572 records were screened based on titles and abstracts. Eighteen full-text articles were assessed for eligibility, of which 15 were excluded. Three studies met the inclusion criteria. Inter-reviewer agreement was assessed qualitatively through discussion. Disagreements were resolved by consensus or, when necessary, by consultation with a third reviewer (A.C). The stages of the search and selection process are summarized in the PRISMA flow diagram (Figure 1).

Instead, studies that did not meet the inclusion criteria were excluded, and the corresponding reasons for exclusion are reported in Table 1.

### 2.5. Data Collection Process

To ensure consistency in data collection, a standardized data extraction sheet was developed using Microsoft Excel (Microsoft Corporation, version 16.100.3, Redmond, WA, USA). The following data were extracted from each included study: authors, country, study design, sample size (female and male), patients’ ages, type of orthodontic treatment (fixed appliance, functional appliance, clear aligner), treatment duration, type of biological sample (saliva or plaque), personnel responsible for biological sample collection, sampling procedures, sample storage conditions, timing of sample collection, and oral health protocols. In addition, two supplementary tables were created: one describing the experimental techniques used for microbial DNA analysis and another detailing the metabolomic analytical methods employed. When results were unclear or missing, data were extracted as reported in the original studies without additional assumptions or imputations. Microorganisms and metabolites were reported as described in the included studies without further validation by the reviewers and should therefore be interpreted with caution.

### 2.6. Risk of Bias

Two reviewers (M.B. and M.P.) independently assessed the risk of bias, and any discrepancies were resolved by consensus. Two different tools were used: the Risk of Bias in Non-Randomized Studies of Interventions (ROBINS-I) was applied to non-randomized studies, while the Revised Version of Cochrane’s Risk of Bias tool (ROB 2) was used for randomized studies [36,37]. The ROBINS-I tool assessed bias related to confounding, selection of participants, classification of interventions, deviations from intended interventions, missing data, measurement of outcomes, and selection of the reported results. The ROB 2 tool evaluated bias arising from the randomization process, deviations from intended interventions, missing outcome data, measurement of outcomes, and selection of the reported results.

### 2.7. Assessment of Certainty of Evidence

The certainty of the evidence for each outcome was evaluated in accordance with the Grading of Recommendations Assessment, Development and Evaluation (GRADE) framework [38]. The GRADE domains were evaluated by two reviewers (M.B and M.P), and a third co-author (A.C) was involved to resolve any disagreements. The rationale for each judgment is reported in Table 2.

## 3. Results

### 3.1. Study Characteristics of the Studies

The demographic and the methodological characteristics of the studies included in this review are summarized below and in Table 3. All three studies were published between 2023 and 2025 and were conducted in China. A total of 136 patients were enrolled, with a mean age ranging from 13 to 23 years [11,18,39]. Only two studies reported the gender distribution of patients, including 58 males and 58 females, while one study did not provide this information. Two studies compared fixed orthodontic appliances with clear aligners, and only one explicitly described treatment modalities within subgroups (Angel Align aligners, Ormco fixed appliances and no treatment) [11,18]. The third study exclusively investigated patients treated with Invisalign aligners [39]. In two of the included studies, the reported treatment duration exceeded one year [18,39]. The biological samples collected included supragingival plaque from the premolar and molar regions of the fourth quadrant, collected by an orthodontist using a Gracey curette; supragingival plaque from specific dental sites (16, 11, 26, 36 and 46) was collected by a periodontist using sterile cotton swabs; and unstimulated saliva collected by two orthodontists using sterile tubes. The sampling protocol varied among the included studies. Specifically, Gong et al. collected samples between 2:00 and 5:00 p.m., Xie et al. between 8:00 and 9:00 a.m., and Song et al. between 9:30 and 10 a.m. In all studies, patients were instructed to refrain from eating and drinking before sample collection; however, only two studies specified a 20 min rest period prior to sampling [18,39]. Before microbiome and metabolomic analyses, biological samples were stored on ice at −80 °C or in liquid nitrogen to preserve molecular stability and prevent degradation. Only one study investigated temporal changes occurring over the course of treatment [11]. Data regarding the oral hygiene protocols followed by patients were provided in only two of the included studies [18,39]. Overall, the included studies showed comparable methodological approaches, although some differences were observed in sampling protocols, data reporting and duration of follow-up.

### 3.2. Microbiome

The studies included in this systematic review suggested a broadly comparable analytical workflow for oral microbiome profiling including DNA extraction, 16S rRNA gene amplification, sequencing, and bioinformatic analysis, as summarized in Table 4. All studies employed next-generation sequencing techniques (Illumina-USA) to characterize microbial communities associated with orthodontic appliances, followed by taxonomic classification and diversity analyses using established bioinformatic pipelines [12,19,40]. Overall, similar molecular approaches for microbial DNA analysis were reported across the included studies. Commercial DNA extraction Kits (Fast Pure Stool DNA Kit (MJYH, Shanghai, China) Omega Bio-Tek DNA Kit (Omega Bio-Tek, Norcross, GA, USA), Omega Mag-Bind Soil DNA Kit (Omega Bio-tek, Norcross, GA, USA)) were used to analyze the V3–V4 regions of the bacterial 16S rRNA gene using the 338F/806R primer set. Comparable amplification and sequencing protocols were described across studies.

Sequencing data were processed to obtain amplicon sequence variants (ASVs) using the DADA2 pipeline (Divisive Amplicon Denoising Alghoritm), which models sequencing errors to infer exact biological sequences without clustering, in two studies [18,39]. In contrast, Gong et al. reported grouping sequences into operational taxonomic units (OTU) [11]. Taxonomic classification was conducted using different classifiers, including Naive Bayes, VSEARCH H/BLAST, RDP [11,18,39]. The Silva database was used by Xie et al. and Gong et al., whereas Song et al. employed the Greengenes database and FastTree2 [11,18,39]. All included studies reported both alpha and beta diversity metrics to explore variations in the oral microbiome in relation to orthodontic treatment. Regarding alpha diversity, all studies reported differences between groups; however, the findings were heterogeneous and primarily descriptive.

Variations in alpha diversity were reported in patients with poor oral hygiene and in patients without white spot lesions, particularly among those treated with fixed appliances (Ormco, Brea, CA, USA) and clear aligners (Angel Align and Invisalign) [18,39]. Regarding beta diversity, two studies reported no statistically significant differences between aligner-treated and the fixed appliance-treated groups, or between Invisalign-treated patients with and without white spot lesions [11,39]. In contrast, Xie et al. reported that oral microbial diversity varied according to oral health status and orthodontic treatment modality, with patients treated with clear aligners (Angel Align, Shanghai, China) showing lower microbial heterogeneity compared with those treated with fixed appliances (Ormco). The microbial taxa varied across studies. *Lentimicrobium* was reported in untreated healthy patients, whereas *Prevotella*, *Fusobacterium*, *Peptostreptococcus*, *Treponema* and *Tannerella* were described in untreated patients with poor oral hygiene [18]. *Streptococcus* and *Granulicatella* were reported in patients treated with Angel Align aligners and good oral hygiene, while *norank f. norank o*. and *Clostridia UCG-014* were described in Angel Align-treated patients with poor oral hygiene [18]. In patients treated with fixed appliances (Ormco), *Rothia* and *Anaeroglobus* were reported in both good and poor oral hygiene groups. In the study by Song et al., all patients were treated with Invisalign, and microbiome findings were stratified according to the presence or absence of white spot lesions. *Actinobacteria*, *Actynomycetales*, *Micrococcaceas*, *Rothia*, *Subdoligranulum*, *Capnocytophaga*, *Flavobateriaceae*, *Azospira*, *Oslenella*, *ASSO_13*, *Lachnoanaerobaculum*, *Abiotrophia*, *and Xanthomonadaceae* were reported in association with white spot lesions [39]. In contrast, *Firmicutes*, *Cetobacterium* and *Burkholderia* were described in patients without white spot lesions [39]. Gong et al. reported the presence of *Veilonella*, *Mogibacterium*, *unclassified c Actinobacteria*, *Actinomyces massiliensis*, *Prevotella pallens*, and *Prevotella jejuni* in patients treated with fixed appliances, whereas these taxa were described as less abundant in patients with clear aligners [11]. Across the included studies, *Prevotella* and *Rothia* were commonly reported microbial genera [11,18,39].

### 3.3. Metabolome

Two of the included studies employed liquid chromatography coupled with mass spectrometry for metabolomic analysis (LC-MS), using the Human Metabolome database (HDMDB), Metlin and Majorbio for metabolite identification [11,18]. One study adopted ultra-performance liquid chromatography coupled with tandem mass spectrometry (UPLC-MS/MS) with data processing performed using Metabo Analyst 4.0 [39]. A summary of the analytical techniques and databases used is provided in Table 5. Across the three included studies, alterations were reported in several broad classes of metabolites, including amino acids and amino acid derivatives, organic acids, lipid- and fatty acid-related compounds, vitamin- and cofactor-related metabolites, and unclassified substances. Two studies reported differences in the number of altered metabolites between patients treated with clear aligners and those treated with fixed appliances [11,18]. In particular, Xie et al. described differences in metabolite profiles among subgroups with different oral hygiene conditions treated with fixed appliances, clear aligners, or receiving no orthodontic treatment. These findings were reported in relation to oral health status rather than to orthodontic appliance type alone.

Amino acids and amino acid derivatives represented the most frequently reported class of metabolites across studies. These included, among others, asparagine, acid aspartic, acid glutamic, glutamine, glycine, histidine, isoleucine, leucine, lysine, methionine, N phenylacetyl phenylalanine, phenylalanine, pyroglutamic acid, serine, proline, threonine, tryptophan, cholarginine, O acetylserine, L-4 hydroxyglutamate, and gamma glutamyleucine [11,18,39].

Organic acids included aliphatic and aromatic carboxylic acids such as 2-hydroxypentanoic acid, 2 benzothiazolsulfonic acid, dehydroeburicoic acid, 6-(4-9carboxymethylphenoxyl)-3,4,5-thihydroxyoxane-2-carboxilic acid, 4-hydroxybenzoic acid, tartaric acid, azelaic acid, benzoic acid, lactic acid, 2 hydroxybutyric acid, valeric acid, 2 propylpent 3 enoic acid [11,18,39]. In addition, non-organic acid compounds, such as acetophenone, were reported in individual studies [11].

Lipid-related metabolites comprised fatty acids, ceramides, phospholipids and isoprenoid compounds, including Lysope, gamma linoleic acid, Cer (d17:1/6 keto PGF1 alpha) phoenicoxantin. Vitamin- and cofactor-related metabolites, including pteridine derivatives, were also reported and included alpha CHECH glucuronide, riboflavin, 2-amino-4-hydroxy-6,7-dymetil-5,6,7,8-tetrahydropteridine [11,18,39]. With regard to metabolic pathways, one study reported variations in pyrimidine metabolism in patients treated with fixed appliances between baseline (T0, before treatment) and follow-up (T2, ≥6 months). In patients treated with clear aligners, alterations in immune-related pathways and changes in purine metabolism were described. Sustained expression of platelet activation-related pathways was reported at T2, which may reflect persistent inflammatory activity over time [11]. Additional unidentified metabolites including annomuricatin B, phoenicoxantin, and ggstop, were also reported [11,18]. Overall, metabolomic findings were heterogeneous across studies and should be interpreted as descriptive, given the differences in analytical platforms, reporting strategies, and study design.

### 3.4. Microbiome—Metabolome and Orthodontic Treatments

Across the included studies, microbiome–metabolome relationships were descriptively reported in the original articles. Overall, the reported associations were heterogeneous and showed limited overlap across studies. Differences in study design including cross sectional and longitudinal approaches, analytical platforms, and reporting strategies limited comparability and precluded any quantitative synthesis. At descriptive level, the available evidence suggested that variations in oral microbial composition were accompanied by changes in metabolite profiles mainly involving amino acid-related, organic acid-related, lipid-related, and vitamin- or cofactor-related pathways. These observations appeared to vary according to oral hygiene conditions and orthodontic treatment modality rather than following consistent or reproducible patterns across studies. Given the exploratory nature of this systematic review, and the methodological heterogeneity of the available data, no quantitative synthesis was feasible. Accordingly, the microbiome-metabolome findings are presented to document the range of observations reported in the literature rather than to infer mechanistic or causal relationships. Detailed microorganism–metabolite associations reported in the included studies are summarized in Table 6.

### 3.5. Oral Hygiene Protocol

Information regarding oral hygiene practices was variably reported across the included studies [11,18,39]. In the study by Gong et al., oral hygiene information was limited [11]. Xie et al. reported partial data on oral hygiene behaviors, indicating that 31% of patients used electric toothbrushes, 26% reported mouthwash use, and 62% brushed their teeth twice daily [18]. The most detailed description was provided by Song et al. Among patients with white spot lesions, brushing frequency of one to two times per day, brushing duration of ≤2 min, limited use of adjunctive oral hygiene aids, and minimal use of dental floss were reported. In contrast, patients without white spot lesions more frequently reported brushing three or more times per day, although brushing duration remained ≤2 min in a substantial proportion of participants. Dietary-related behaviors, including frequent consumption of carbonated soft drinks, were also reported and differed between groups [39]. Overall, heterogeneity in the reporting of oral hygiene practices represents a potential source of confounding for both microbiome and metabolomic outcomes and should be considered when interpreting the findings.

### 3.6. Risk of Bias

**Figure 2:**
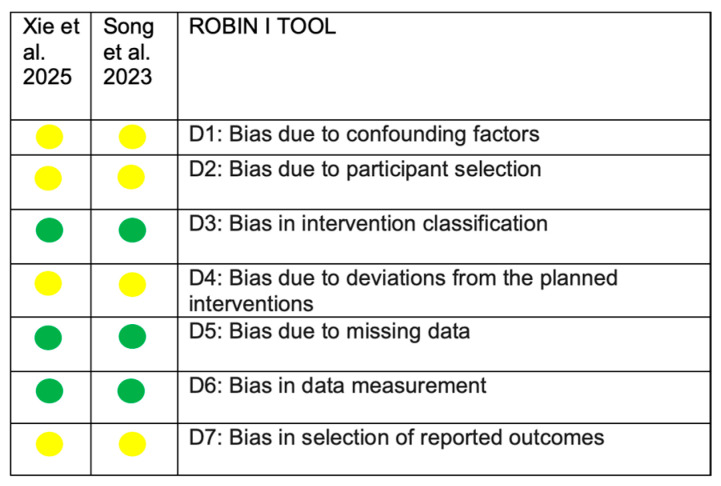
ROBINS I tool. Green circles indicate a low risk of bias, whereas yellow circles indicate a moderate risk of bias.

**Figure 3:**
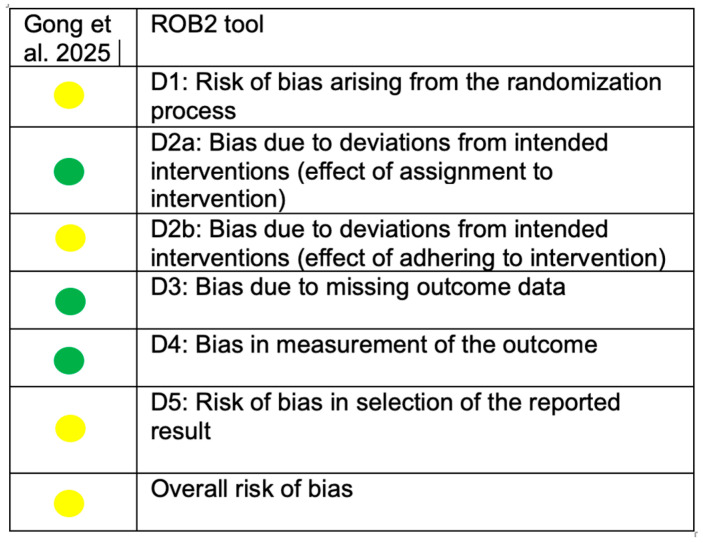
ROB 2 tool. Green circles indicate a low risk of bias, whereas yellow circles indicate a moderate risk of bias.

A summary of the overall risk of bias assessment for the included studies is presented in Figure 2 and Figure 3. For the two cross-sectional studies, the ROBINS-I tool was applied, as group allocation was not based on true randomization but rather on pre-existing clinical characteristics, including the type of orthodontic treatment, oral health status, and the presence or absence of white spot lesions [18,39]. The revised version of Cochrane’s Risk of Bias tool (ROB-2) was used for the randomized study [11]. Overall, all three studies were judged to have a moderate risk of bias, mainly related to potential confounding and participant selection. Group formation based on clinical characteristics may have introduced residual confounding, although inclusion criteria were clearly defined. Some concerns were also identified regarding deviations from the intended interventions, as both patients and clinicians were aware of the treatment received, and potential co-interventions such as diet and oral hygiene practices could not be fully controlled. No major concerns were identified regarding outcome reporting. Domains assessed as having a low risk of bias were not further discussed.

### 3.7. Strength of Evidence

The quality of the evidence was assessed using the Grading of Recommendations Assessment, Development and Evaluation (GRADE) approach, considering study design, risk of bias, inconsistency, indirectness, imprecision and publication bias across the included studies (Table 2).

Regarding study design, the certainty of evidence was rated as moderate. Two studies were non-randomized but followed clearly defined clinical protocols, while the third study had a prospective design and was downgraded due to the lack of a detailed description of the randomization procedure. The risk of bias domain was judged as moderate, based on the overall methodological quality of the studies. Inconsistency was rated as serious for metabolomic and oral hygiene outcomes in orthodontic patients, as the reported results showed heterogeneity and divergence across the three included studies, despite clinically well-defined study designs. Indirectness and publication bias were not considered serious. Imprecision was downgraded and judged as serious for metabolomic and oral health outcomes due to the limited amount of heterogeneous evidence, reflecting the constraints of the available data.

## 4. Discussion

In recent years, while several studies have focused on the microbiological aspects of oral health, increasing attention has been directed toward the potential contribution of metabolomic analyses to improve the understanding of biological changes and their possible role in informing future personalized treatment strategies. This systematic review identifies gaps in the current literature and examines the available evidence on metabolomic alterations in orthodontic settings. The included studies primarily involved young patients treated with fixed appliances or clear aligners. Demographic factors, particularly age and gender, may influence metabolic profiles through hormonal fluctuations involving estrogen, androgens and cortisol which are known to affect lipid metabolism, inflammatory responses, and the oxidative status of the oral mucosa [40,41]. Lifestyle-related factors, especially dietary habits, may further contribute to variability in both microbiome composition and metabolomic profiles [42,43]. As all included studies were conducted in China, population-specific lifestyle behaviors and dietary patterns may have influenced the observed findings. Consequently, the reported metabolic patterns may partially reflect the population—specific characteristics, which should be considered when interpreting the results. In this context, general health status may represent an additional confounding factor. For instance, a meta-analysis reported an association between type 2 diabetes risk and altered plasma amino acids profiles, including increased levels of leucine, valine, tyrosine and phenylalanine, together with reduced glycine and glutamine concentrations [44]. From a methodological perspective, heterogeneity in the type of biological samples analyzed may have influenced the reported metabolomic findings. Supragingival dental plaque and saliva represent distinct biological matrices with specific characteristics. Saliva is a predominantly aqueous biofluid whose molecular composition varies in response to physiological and pathological conditions [45]. Conversely, supragingival dental plaque represents a site-specific microbial biofilm closely associated with tooth surfaces and local metabolic activity, and its composition is strongly influenced by individually tailored instruction oral hygiene practices [46].

Beyond patient-related and methodological factors, the type of orthodontic appliance may represent an additional element influencing the oral environment. Less favorable periodontal clinical parameters have been reported in patients treated with fixed appliances, potentially related to increased plaque retention and reduced effectiveness of oral hygiene measures. In contrast, clear aligners have been increasingly adopted because of their association with improved oral hygiene and periodontal health [47,48,49,50]. However, clear aligner therapy requires prolonged daily wear, often up to 22 h, resulting in continuous coverage of tooth surfaces, which may reduce the protective buffering effect of saliva [51]. Moreover, behavioral habits such as eating and drinking while wearing aligners may further modify the oral microenvironment, potentially contributing to metabolic imbalance. Consistent with these observations, recent studies have reported a higher incidence of white spot lesions in patients treated with aligners [52,53]. In addition, stainless steel brackets and polymer-based clear aligners differ in surface characteristics, physiochemical properties and interactions with saliva and oral biofilms. These material-related differences may influence local biological processes and could contribute to distinct biological responses, despite generally favorable clinical outcomes [54]. Taken together, these observations suggest that fixed appliances and clear aligners may influence the oral microenvironment through different mechanisms, potentially contributing to distinct local biological and metabolomic responses. The findings of this review indicate that certain metabolomic patterns observed during orthodontic treatment may be associated with localized oral changes. In particular, during clear aligner therapy, variations in amino acids such as glutamine, proline, glycine and serine have been reported. Proline is involved in collagen metabolism and connective tissue turnover while glycine contributes to glutathione synthesis and cellular antioxidant mechanisms, participating in pathways related to protein turnover and cellular homeostasis [55]. The detection of these amino acids may reflect metabolic adaptations occurring in the periodontal tissues during prolonged aligner wear, which can modify the oral microenvironment. Such changes may contribute to conditions permissive to enamel demineralization rather than representing direct pathogenic mechanisms. Previous studies have suggested that increased proline levels may be associated with salivary proline-rich proteins, which are more susceptible to proteolytic degradation and may serve as indirect indicators of altered salivary–microbial interactions [56]. Other amino acids reported across the included studies, including glutamine, serine, valine and leucine, are known to support immune cell metabolism and protein synthesis [57]. Variations in their levels may therefore reflect tissue stress or adaptive responses to orthodontic forces, potentially associated with redox imbalance and shifts in the oral microbial ecosystem, rather than overt disease processes. Such modifications of the oral environment may influence the dynamic balance between demineralization and remineralization [58,59]. Associations between amino acids profiles and microorganisms such as *Lachnoanareobaculum*, *Rothia* and *Subdoligranulum* suggest coordinated microbiome-metabolome interactions; however, these relationships should be interpreted cautiously, given the limited number of studies and substantial heterogeneity. Similarly, metabolites including L-4 hydroxy glutamate and γ glutamyl leucine have been associated with redox-related processes, while alpha CHEC glucuronide, adenine, and riboflavin may reflect increased metabolic activity and cellular turnover under inflammatory conditions [58]. Acidic metabolites derived from microbial fermentation pathways, including 2-hydroxypentanoic acid and 5-aminopentanoic acid, have been associated with *Streptococcus*, *Actynomices*, and *Prevotella*, microorganisms linked to acidogenic activity and caries-associated demineralization [60]. In contrast, lipid-related metabolites such as Lysope and lysophospolipids may represent nonspecific markers of membrane turnover or tissue damage, consistent with low grade inflammatory responses observed during orthodontic treatment, particularly in patients with fixed appliances, indicating an increased susceptibility to periodontal disease. Their presence has been correlated with *Anaeroglobus* and *Prevotella*. Moreover, 2-propylpent-3-enoic acid, a metabolite associated with lipid peroxidation, further supporting the inflammatory activity [60,61]. Finally, although clear aligners are generally associated with favorable clinical outcomes, one study reported indicators of increased immune activity and platelet responsiveness. The detection of unclassified compounds such as Annomuricatin B, ggstop and phoenicoxantin, instead should therefore be interpreted with caution, as they may reflect analytical inference or exogenous sources rather than endogenous metabolic activity [62]. From a clinical perspective, the integration of metabolomic and microbiological analyses may support hypothesis generation regarding biological processes occurring during orthodontic treatment. The identification of specific metabolites may reflect biological pathways potentially influenced by orthodontic appliances, as well as by patient-related conditions and exogenous factors. At present, these observations remain exploratory and are not intended to guide clinical decision-making; however, they may inform future research aimed at advancing personalized approaches in orthodontics.

## 5. Conclusions

Overall, this review aimed to describe preliminary metabolomic patterns potentially associated with orthodontic treatments. The integration of metabolomic and microbiological analyses may help support hypothesis generation regarding biological processes occurring during orthodontic treatment. The identification of specific metabolites suggests potential biological pathways that may be influenced by orthodontic appliances, as well as by systemic conditions and exogenous factors such as lifestyle. Further research based on standardized protocols and longitudinal study designs is required to clarify the clinical findings of these observations and to explore their potential role in personalized monitoring and prevention strategies in orthodontic patients.

## 6. Limitations

Despite the potential promise of metabolomic approaches in orthodontics, this review has several important limitations that should be considered when interpreting the findings. Only three clinical studies, all conducted in a single country and involving small sample sizes, met the inclusion criteria. A meta-analysis was not feasible due to the limited number of studies and substantial heterogeneity of the results. Considerable methodological heterogeneity was observed across the included studies, including differences in study design (cross section versus longitudinal) type of orthodontic appliance, biological samples analyzed (saliva and supragingival plaque), sampling time points, analytical platforms, and metabolite reporting strategies. This heterogeneity limits direct comparability and supports a primarily descriptive and hypothesis-generating interpretation of the findings. Several potential confounding factors were inconsistently reported, particularly oral hygiene practices, dietary habits and sampling timing, which were not systematically controlled across the included studies. Although oral hygiene protocols were summarized where available, incomplete and heterogeneous reporting represents a potential source of bias and precludes causal inference regarding microbiome–metabolome associations during orthodontic treatment. In addition, the overall certainty of evidence was limited, with a moderate risk of bias identified across outcomes. Finally, metabolite identification was reported as described in the original studies, and confidence levels were not uniformly provided, requiring cautious interpretations of some metabolites, particularly those unexpected in human oral samples. Taken together, these limitations highlight the need for future well-designed studies using standardized protocols for sampling, analytical methods, confounder control, and metabolite identification to better clarify the clinical relevance of metabolomic changes during orthodontic treatment.

## Figures and Tables

**Table 1:** Excluded articles with reasons.

Authors	Reasons for Exclusion
Zheng et al. [22]	No actual metabolomic analysis was performed. Only a general mention was provided, without a specific metabolite characterization; the analysis focused primarily on the oral microbiome.
Da Costa Rosa et al. [23]	An ex vivo model was used to investigate caries progression/arrest on extracted premolars, with no evaluation of the effects of orthodontic treatment on the oral metabolome.
Gao et al. [3]	The study evaluated oral microbiome and salivary pH changes in two groups treated with thermoplastic polyurethane (TPU)and polyethylene terephthalate glycol (PETG)aligners. No metabolomic profiling was performed.
Liu et al. [24]	No metabolomic profiling was performed.
Yang et al. [25]	The study included groups of periodontally compromised patients, and the reported metabolomic analysis was based on predictive models rather than direct metabolomic measurements.
Shintani et al. [26]	Salivary microbiome analysis (16S rRNA sequencing) without orthodontic intervention; no direct metabolomic profiling was performed.
Chen et al. [27]	Microbiological analysis only (functional appliances vs. clear aligners); no metabolomic profiling was performed
Nemec et al. [28]	The study focused on the salivary microbiome (16S rRNA sequencing) and inflammatory markers (MRP-8/14); no metabolomic profiling was performed.
Yang et al. [29]	No metabolomic profiling was performed.
Zhao et al. [30]	No metabolomic profiling was performed.
Miao et al. [31]	No metabolomic profiling was performed.
AlShahrani et al. [32]	No metabolomic profiling was performed.
Vrankova et al. [33]	The study focused on tongue microbiota in relation to breathing mode (nasal versus mouth breathing) in children undergoing orthodontic treatment; no metabolomic analysis was performed.
Wang et al. [34]	Metagenomic analyses inferring metabolic pathways (KEGG) were conducted without direct metabolomic measurements (LC-MS, GC-MS, NMR).
Wang et al. [35]	Metagenomic analyses inferring metabolic pathways (KEGG) were conducted without direct metabolomic measurements (LC-MS, GC-MS, NMR).

**Table 2:** GRADE assessment.

Outcome of Interest	Study Design	Risk of BIAS	Inconsistency	Indirectness	Imprecision Results	Publication Bias
Microbiome analysis (fixed appliances vs. clear aligners vs. no treatments	2 cross sectional study, 1 prospective observational study; Moderate *	Moderate ^a^	Not serious ^b^	Not Serious ^e^	Not Serious ^f^	Not Serious ^i^
Metabolome analysis (fixed appliances vs. clear aligners vs. no treatments	2 cross sectional study, 1 prospective observational study; Moderate *	Moderate ^a^	Serious ^c^	Not Serious ^e^	Serious ^g^	Not serious ^i^
Oral health (fixed appliances vs. clear aligners vs. no treatments)	2 cross sectional study, 1 prospective observational study; Moderate *	Moderate ^a^	Serious ^d^	Not Serious ^e^	Serious ^h^	Not serious ^i^

* True randomization was not performed in two of the included studies, although participant selection followed clearly defined clinical criteria. In the prospective study, the randomization procedure was not described in detail. a: Due to the lack of randomization, despite the presence of clearly defined clinical protocols. b: All included studies provided a clear description of the microbiome analyses performed in the different study groups. c: For metabolomic analyses, inconsistency was considered serious due to divergent results and differences in metabolite reporting across studies. d: Oral hygiene was assessed in only two of the included studies, which reported data in separate tables and compared the observed differences; in contrast, one study did not provide this information. e: The included studies met the PICO criteria and directly addressed the research question in terms of population, interventions, comparisons, and outcomes. f: Microbiome analysis results were clearly reported and were generally consistent across studies. g: Regarding metabolomic outcomes, imprecision was rated as serious because one study did not clearly explain or quantify the evidence supporting the higher presence of metabolites in the clear aligner group. h: One of the studies did not take this aspect into account, despite its fundamental importance for the analyses conducted. i: There was no indication of omitted studies or selective reporting of negative results.

**Table 3:** Included studies and data extraction sheet. FA (fixed appliance), CA (clear aligner); AA (Angel Aligners), I (Invisalign), NT (no treatments) GOH (good oral hygiene), POH (poor oral hygiene), SP (supra gingival plaque) US (unstimulated saliva), PBS (1 mL of sterile phosphate-buffered saline). LN_2_ (liquid nitrogen) T0 (before treatment), T1 (1–3 months), T2 (≥6 months), WSLs (white spot lesions), NoWSLs (no presence of white spot lesions), ETB (electric tooth brushing), DBF (daily brushing frequency) M (mouthwash) BD (brushing duration), OTB (orthodontic tooth brush), TF (toothpaste fluoridated), ECA (eating while wearing clear aligners), CSD (prefer frequently carbonated soft drink), NECA (never eating while wearing clear aligners).

Authors	Country	Type of 2025	Sample	Age	Type of Orthodontic Treatments	Treatment Duration	Type of Biological Sample	Operator	Sampling Technique	Sample Storage	Sample Collection Protocol	Collection Time Point	Oral Health Protocol
Gong et al. 2025 [11]	China	Prospective observational study	N = 20; /	22.00 ± 6.99 years	10 FA; 10 CA.	/	SP (premolar-molar area, IV quadrant)	1 orthodontist	Gracey curettes pooled in PBS	ice; −80 °C (30 min)	14:00–17:00 p.m; No food/drink	T0: 10 FA; 10 CA. T1: 7 FA; 8 CA. T2:9 FA; 8 CA.	Collected ≥ 1 h after oral hygiene.
Xie et al. 2025 [18]	China	Cross sectional study	N = 61. (36 F, 25 M)	23 years	10 NT (GOH); 10 NT (POR); 10 CA (AA) GOH; 10 CA (AA) POH; 10 FA (Ormco)-GOH; 11 FA (Ormco)-POH	≥1 years	SP (16, 11, 26, 36, 31,46)	1 periodontist	sterile cotton swabs	LN_2_; −80 °C	8:00–9:00 a.m; No food/drink; rest (20 min) before sampling	T0 = 61	31% (ETB);26% (M),62% (2 DBF)
Song et al. 2023 [39]	China	Cross sectional study	N = 55 (33 M; 22 F)	13.4 ± 2.0 years	55 CA(I) 27 WSLs; 28 NO-WSLs.	≥1 years	US (5 mL)	2 orthodontists	sterile centrifuge tubes	LN_2_; −80 °C	9.30–10:00 a.m.; No food/drink; rest (20 min) before sampling	T0 (55)	WSLs group: 51.9% (1–2 DBF); 81.5% (≤2 BD), 7.4% (M), 11.1% (OTB), 96,3% (no dental floss), 11.1% (TF) 48.1% (ECA), 51.9% (CSD). NoWSLs: 53.6% (≥3 DBF), 60.7% (≤2 min BD), 17.9% (M), 17.9% (OTB), 89.3% (no dental floss), 39.3% (TF), 42.9% (NECA), 42.9% (CSD)

**Table 4:** DNA extraction and procedures of sequencing and processing the DNA with the relative database used by the authors.

Author/Years	DNA eA2.	PCR/Sequencing	Processing/OTU/ASV	Taxonomy and Databases
Xie et al. 2025 [18]	FastPure Stool DNA kit (MJYK, Shanghai, China), quality assessed by 1.0% agarose gel electrophoresis and Nanodrop ND-2000 spectrophotometer (Thermo Scientific, Waltham, MA, USA)	16S rRNA V3–V4(primers 338F/806R) on T100 Thermal Cycler (Bio-Rad, Hercules, CA, USA); PCR mix (Fast Pfu buffer, dNTPs, primers, Fast Pfu polymerase, ddH_2_O); PCR purification (Synergy HTX microplate; Bioterk, Winooski, VT, USA), Illumina NextSeq 2000 PE300 (San Diego, CA, USA)	fastp v0.19.6 (quality filter), FLASH v1.2.11 (merge reads), DADA2 plugin in QIIME2 v2020.2 (ASVs); Good’s coverage = 97.9%)	Naive Bayes (or VSEARCH/BLAST) consensus classifier in QIIME2, using SILVA 16S rRNA v138
Gong et al. 2025 [11]	Omega Bio-tek kit (Omega Bio-tek, Norcross, GA, USA); lysozyme (50 mg/mL, 30 °C, 10 min), proteinase K (0.25 mg/mL, 55 °C, 1 h); centrifugation (10,000× *g*, 5 min)	16S rRNA V3–V4 (primers 338F/806R); PCR purification (Axygen kit); Illumina MiSeq PE300 (Illumin, Inc., San Diego, CA, USA)	fastp v0.19.6 (quality filter), FLASH v1.2.7 (merge reads), UPARSE 7.1 (OTU clustering at 97%)	RDP Classifier v2.2, SILVA v138
Song et al. 2023 [39]	Omega Mag-bind soil DNA kit (Norcross, GA, USA); quality assessed by agarose gel and Nanodrop 2000 (Thermo Scientific, USA); purification by VAHTSTM DNA Clean Beads (Vazyme, Nanjing, China); quantification with Quant-it Pico Green (Invitrogen, Waltham, MA, USA)	16S rRNA V3–V4 (primers 338F/806R); Illumina Novaseq	DADA2 via QIIME2 v2019.4 (ASVs)	FastTree2 and Greengenes database

**Table 5:** Metabolomic analysis with different procedures, namely targeted or untargeted metabolomic processing, respectively, with ultra performance liquid chromatography and tandem mass spectrometry (UPLC MS/MS) and liquid chromatography with tandem mass spectrometry (LC MS/MS) and the relative databases used for the identification of the metabolites.

Author/Years	Metabolomic Technique	Extraction	Databases
Xie et al. 2025 [18]	Untargeted LC–MS/MS (UHPLC-Q Exactive HF-X system), DDA acquisition, Majorbio, Shanghai, China)	50 mg plaque + methanol:water (4:1) + 0.02 mg/mL L-2-chlorophenylalanine → homogenization, ultrasonication (40 kHz, 5 °C, 30 min), incubation (−20 °C, 30 min), centrifugation (13,000× *g*, 4 °C, 15 min).	HMDB, Metlin, Majorbio
Gong et al. 2025 [11]	Untargeted LC–MS/MS (UHPLC-Q Exactive HF-X system)	acetonitrile:methanol (1:1) + 0.02 mg/mL L-2-chlorophenylalanine → protein precipitation; supernatant re-solubilized (acetonitrile:water), ultrasonicated (40 kHz, 5 °C, 5 min), centrifuged (13,000× *g*, 4 °C, 10 min)	HMDB, Metlin, Majorbio
Song et al. 2023 [39]	Targeted UPLC–MS/MS	Saliva (100 µL) lyophilized → reconstituted (with 20 µL of 50% methanol). Automated handling (Eppendorf epMotion, Hamburg, Germany): add 120 µL ice-cold methanol + internal standards, vortex 5 min, centrifuge (4000 g, 30 min, 4 °C).Derivatization: +20 µL reagents, 30 °C × 60 min → dilute 330 µL 50% methanol, centrifuge (4000× *g*, 30 min 4 °C); transfer 135 µL supernatant (with 10 µL internal standards) to new plate.	MetaboAnalyst 4.0

**Table 6:** Associations are reported as described in individual studies and may reflect study-specific co-occurrence patterns rather than consistent or causal relationships.

Microorganisms	Metabolites	Orthodontic Treatment Context (as Reported)
*Anaeroglobus*	alpha CEHC glucuronide, Lysope	Fixed appliances
*Rothia*	glycine, proline, glutamine, serine, lactate	Clear aligners, Fixed appliances.
*Rothia*	annomuricatin B	Clear aligners
*Prevotella*	2 hydoxypentanoic acid, Lysope	Fixed appliances
*Prevotella*	Acetophenone, ggstop, 2 propylpent 3 enoic acid, adenine, riboflavin, O acetylserine, L-4 hydroxyglutamate, and gamma glutamyleucine	Fixed appliances, Clear aligners
*Actynomices*	leucine and valine	Fixed appliances, Clear aligners
*Subdolinogranulum* and *Lachnoanaerobaculum*	glycine, proline, glutamine, serine, lactate	Clear aligners

## Data Availability

No new data were created or analyzed in this study. Data sharing is not applicable to this article.

## Supplementary Materials

The following supporting information can be downloaded at: https://www.mdpi.com/article/10.3390/medicina62010224/s1: PRISMA 2020 checklist.
